# TMEM65 promotes gastric tumorigenesis by targeting YWHAZ to activate PI3K-Akt-mTOR pathway and is a therapeutic target

**DOI:** 10.1038/s41388-024-02959-9

**Published:** 2024-02-10

**Authors:** Lingxue Shi, Xiaohong Wang, Shang Guo, Hongyan Gou, Haiyun Shang, Xiaojia Jiang, Chunxian Wei, Jia Wang, Chao Li, Lihong Wang, Zengren Zhao, Weifang Yu, Jun Yu

**Affiliations:** 1 Departments of Endoscopy Center, The First Hospital of Hebei Medical University, Shijiazhuang, China; 2 Institute of Digestive Disease and Department of Medicine and Therapeutics, State Key Laboratory of Digestive Disease, Li Ka Shing Institute of Health Sciences, Chinese University of Hong Kong-Shenzhen Research Institute, The Chinese University of Hong Kong, Hong Kong SAR, China; 3 The First Hospital of Hebei Medical University, Shijiazhuang, China; 4 Key Laboratory of Carcinogenesis and Translational Research, Peking University Cancer Hospital and Institute, Beijing, China; 5 Gastrointestinal Disease Centre, Hebei Key Laboratory of Colorectal Cancer Precision Diagnosis and Treatment, The First Hospital of Hebei Medical University, Shijiazhuang, China

**Keywords:** Cell biology, Cancer genetics

## Abstract

Copy number alterations are crucial for the development of gastric cancer (GC). Here, we identified Transmembrane Protein 65 (TMEM65) amplification by genomic hybridization microarray to profile copy-number variations in GC. TMEM65 mRNA level was significantly up-regulated in GC compared to adjacent normal tissues, and was positively associated with TMEM65 amplification. High TMEM65 expression or DNA copy number predicts poor prognosis (*P* < 0.05) in GC. Furtherly, GC patients with TMEM65 amplification (*n* = 129) or overexpression (*n* = 78) significantly associated with shortened survival. Ectopic expression of TMEM65 significantly promoted cell proliferation, cell cycle progression and cell migration/invasion ability, but inhibited apoptosis (all *P* < 0.05). Conversely, silencing of TMEM65 in GC cells showed opposite abilities on cell function in vitro and suppressed tumor growth and lung metastasis in vivo (all *P* < 0.01). Moreover, TMEM65 depletion by VNP-encapsulated TMEM65-siRNA significantly suppressed tumor growth in subcutaneous xenograft model. Mechanistically, TMEM65 exerted oncogenic effects through activating PI3K-Akt-mTOR signaling pathway, as evidenced of increased expression of key regulators (p-Akt, p-GSK-3β, p-mTOR) by Western blot. YWHAZ (Tyrosine 3-Monooxygenase/Tryptophan 5-Monooxygenase) was identified as a direct downstream effector of TMEM65. Direct binding of TMEM65 with YWHAZ in the cytoplasm inhibited ubiquitin-mediated degradation of YWHAZ. Moreover, oncogenic effect of TMEM65 was partly dependent on YWHAZ. In conclusion, TMEM65 promotes gastric tumorigenesis by activating PI3K-Akt-mTOR signaling via cooperating with YWHAZ. TMEM65 overexpression may serve as an independent new biomarker and is a therapeutic target in GC.

## Introduction

Gastric cancer (GC) ranks fifth in incidence and fourth in mortality worldwide [1]. GC is a heterogeneous disease, which is associated with somatic copy number aberrations (CNAs), genetic alteration, epigenetic changes and chromatin remodeling [2]. Although great progress has been made in the identification and treatment of GC, the incidence and mortality of GC remains high, and the mechanisms of GC are still largely unclear. Studies has been shown that accumulation of DNA copy number is associated with GC pathogenesis and predicts poor prognosis [3]. Thus, it is important to identify the relationship between DNA copy number and genes in cancer initiation and progression of GC, so as to identify the new therapeutic target for GC.

Chromosome 8q24 is a susceptibility locus for multiple cancers, including GC. Amplification of genes on chromosome 8q24 showed poor prognosis in GC patients [4, 5]. TMEM65, located at chromosome 8q24.13, is an integrated membrane protein localized in the inner mitochondrial membrane, which plays a central role in regulating cell function, metabolism and cell death during carcinogenesis [6]. TMEM65 mutation caused mitochondrial dysfunction, increased ROS and resulted in a variety of disease [6–8]. However, the functional significance of TMEM65 in GC has not been explored.

By analyzing The Cancer Genome Atlas (TCGA) database, we found that increased TMEM65 DNA copy number was associated with its mRNA expression in GC. Therefore, we hypothesized that TMEM65 overexpression due to its copy number gain is involved in gastric carcinogenesis [9]. Indeed, we demonstrated that TMEM65 is universally overexpressed in GC patients and is significantly associated with poor survival of GC patients. In this study, we investigated the functional role, molecular mechanism and clinical implication of TMEM65 in gastric carcinogenesis.

## Results

### TMEM65 is upregulated in GC patients and is associated with poor survival

**Fig. 1:**
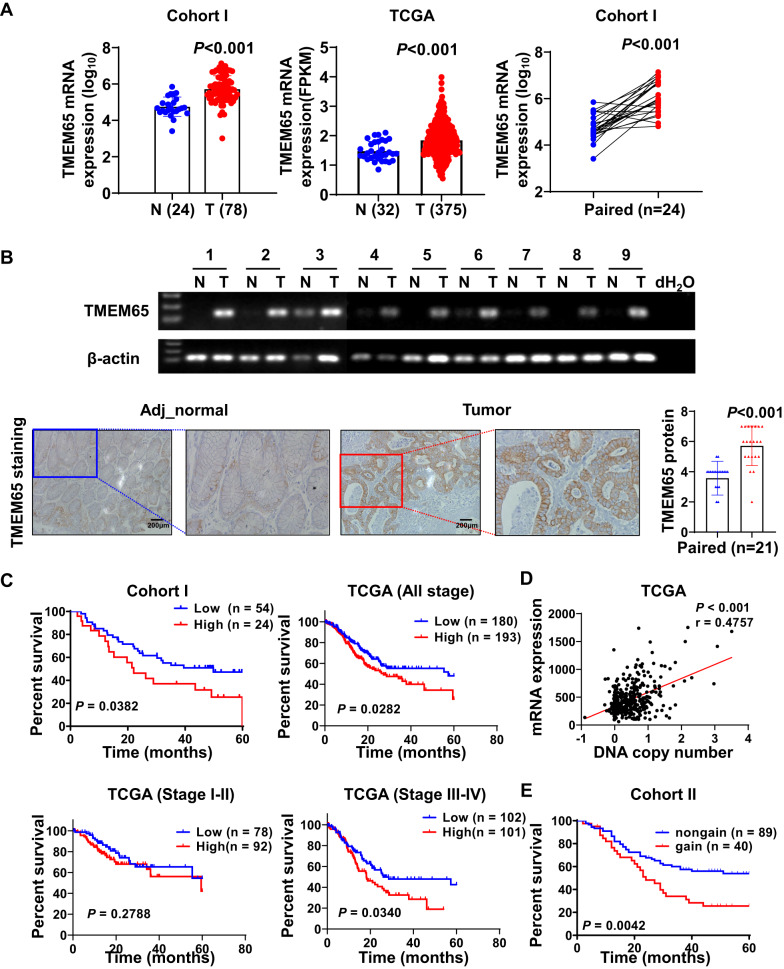
TMEM65 is upregulated in GC patients and predicts poor survival. **A** TMEM65 mRNA expression was upregulated in GC tissues as compared with normal tissues in cohort I and TCGA cohort, and furtherly confirmed in paired GC tumor tissues from cohort I. **B** TMEM65 mRNA expression was upregulated in GC compared with paired adjacent normal tissues, as shown by regular RT-PCR, and TMEM65 protein expression was upregulated in GC tumor tissues than that in adjacent normal tissues as determined by IHC. **C** Kaplan–Meier survival analysis from cohort I showed GC patients with high TMEM65 expression had poorer survival. Kaplan–Meier survival analysis from TCGA cohort showed that GC patients with high TMEM65 expression (all stage, stage III-IV) had poorer survival compared with GC patients with low TMEM65 expression. **D** TCGA cohort showed a positive correlation between DNA copy number and TMEM65 mRNA expression level. **E** Kaplan–Meier survival analysis from cohort II showed GC patients with high TMEM65 DNA copy number had poorer survival than those with low TMEM65 DNA copy number.

We examined TMEM65 expression in GC and normal tissues. TMEM65 mRNA was significantly overexpressed in GC tissues as compared with normal tissues in cohort I by Affymetrix chip assay (adjacent normal, *n* = 24; GC, *n* = 78; *P* < 0.001) and TCGA cohort by RNA-seq and normalized as Fragments Per Kilobase of transcript per Million mapped reads (FPKM) (adjacent normal, *n* = 32; GC, *n* = 375; *P* < 0.001), and was confirmed in paired GC tumor tissues as compared to adjacent normal controls in cohort I (*n* = 24; *P* < 0.001) (Fig. 1A). We verified TMEM65 mRNA expression was significantly overexpressed in GC tumors as compared to adjacent normal tissues by regular RT-PCR (Fig. 1B). Moreover, the protein level of TMEM65 in GC tissues was much higher than adjacent normal tissues by immunohistochemistry staining (IHC) (*P* < 0.001; Fig. 1B). As shown in the Kaplan–Meier survival curves, patients with high TMEM65 expression in cohort I showed lower survival rate than those with low TMEM65 expression (*P* < 0.05). Consistently, GC patients with high TMEM65 expression showed poor survival from TCGA cohort (*P* < 0.05). After stratification by tumor staging, high TMEM65 predicted poor prognosis in stage III-IV GC patients (*P* < 0.05; Fig. 1C) but not in stage I-II patients (Fig. 1C). We further evaluated the clinicopathologic significance of TMEM65 expression in GC patients. TMEM65 overexpression was associated with an increased risk of cancer-related death by univariate Cox regression analysis [HR = 2.028, 95% CI (1.709–3.813); *P* < 0.05; Table 1]. In particular, after adjustment for potential confounding factors, TMEM65 expression was a predictive factor for poor survival independent of Tumor-Nodes-Metastasis (TNM) stage in patients with GC by Multivariate Cox regression analysis [HR = 2.001, 95% CI (1.055–3.793); *P* < 0.05; Table 1]. These findings indicated that TMEM65 predicts poor prognosis in GC patients. Furthermore, we found that TMEM65 mRNA expression level was positively correlated with DNA copy number gain (*P* < 0.001; R = 0.4757; Fig. 1D), GC patients with high DNA copy number had poor survival in cohort II (*n* = 129, *P* < 0.05; Fig. 1E). These findings collectively indicated that TMEM65 is commonly overexpressed due to copy number gain and may serve as a new independent biomarker for the prognosis of GC patients.

**Table 1:** Univariate and Multivariate Cox regression analysis of potential poor prognostic factors for GC patients.

Variables	Univariate Cox regression analysis	
	HR (95%CI)	*P* value
**Age** (>60 *vs*. ≤60)	1.056 (0.559–1.995)	0.866
**Gender** (Male *vs*. Female)	0.557 (0.256–1.209)	0.139
**TMEM65 expression** (High *vs*. Low)	2.028 (1.709–3.813)	*P* < 0.05
**Location** (Cardia *vs*. non-cardia)	0.475 (0.254–0.888)	*P* < 0.05
**Differentiation** (Low *vs*. High and moderate)	1.114 (0.493–2.515)	0.795
**T stage (**III and IV *vs*. I and II)	1.090 (0.336–3.540)	0.885
**N stage** (Positive *vs*. Negative)	2.507 (0.889–7.068)	0.082
**M stage** (Positive *vs*. Negative)	4.941 (2.251–10.846)	*P* < 0.0001
**TNM stage** (III and IV *vs*. I and II)	3.769 (1.457–9.752)	*P* < 0.01
**Variables**	**Multivariate Cox regression analysis**	
	**HR (95%CI)**	***P*** **value**
**Age** (>60 *vs*. ≤60)	1.207 (0.622–2.343)	0.579
**Gender** (Male *vs*. Female)	0.569 (0.247–1.311)	0.185
**TMEM65 expression** (High *vs*. Low)	2.001 (1.055–3.793)	*P* < 0.05
**Differentiation** (Low *vs*. High and moderate)	1.103 (0.453–2.687)	0.828
**TNM stage** (III/IV *vs*. I/II)	3.559 (1.352–9.371)	*P* < 0.05

### TMEM65 promotes GC cells growth in vitro

**Fig. 2:**
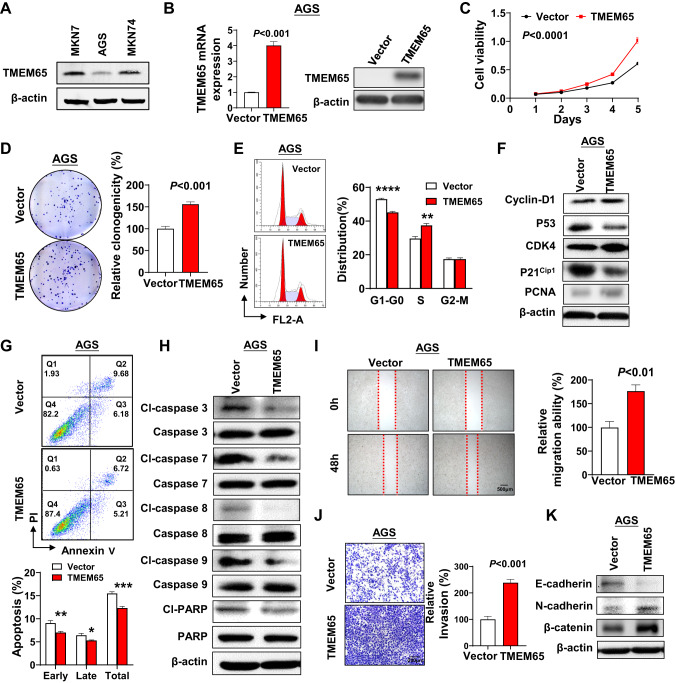
TMEM65 promotes GC cell growth, migration and invasion in vitro. **A** The expression of TMEM65 in GC cell lines was determined by Western blot. **B** Ectopic expression of TMEM65 in AGS cells was confirmed by qRT-PCR and Western blot. **C**, **D** Ectopic expression of TMEM65 in AGS cells significantly promotes cell viability **C** and cell clonogenicity **D**. **E** Ectopic expression of TMEM65 in AGS cells obviously accelerated G1-S cell cycle progression, determined by flow cytometry. **F** Western blot showed overexpression of TMEM65 increased the protein expression of Cyclin-D1, CDK4, PCNA and reduced the expression of P53, P21^cip1^. **G** Ectopic expression of TMEM65 in AGS cells inhibited early and late apoptosis, determined by flow cytometry. **H** Western blot showed ectopic expression of TMEM65 in AGS cells reduced the active expression levels of cleaved-caspase-3, cleaved-caspase-7, cleaved-caspase-8, cleaved-caspase-9, and cleaved-PARP. **I** Representative images of wound healing assay revealed that ectopic expression of TMEM65 promoted cell migration in AGS cells. **J** Representative images of Matrigel invasion transwell assay revealed that ectopic expression of TMEM65 promoted cell invasion in AGS cells. **K** Western blot showed ectopic expression of TMEM65 increased the protein expression of N-cadherin, β-catenin, and decreased the expression of E-cadherin.

We then examined the protein expression of TMEM65 in three GC cell lines (Fig. 2A). Among them, two GC cells (MKN7, MKN74) had relatively high expression of TMEM65, and AGS had low expression. The result of TMEM65 upregulated in GC prompted us to verify its potential oncogenic role. We used a TMEM65-expressing lentivirus to construct AGS stable expressing TMEM65 cells. Ectopic expression of TMEM65 in AGS cells was examined by qRT-PCR and Western blot (Fig. 2B). Cell viability (Fig. 2C**)** and colony formation (Fig. 2D**)** assays showed that overexpression of TMEM65 in AGS cells promoted cell proliferation (*P* < 0.0001) and colony formation ability (*P* < 0.001). These results indicated that TMEM65 plays an oncogenic role in GC.

Ectopic expression of TMEM65 accelerated G1-S cell cycle progression as determined by flow cytometry in AGS cells (*P* < 0.01; Fig. 2E). Consistently, TMEM65 increased the protein expression of cell cycle related markers Cyclin-D1, CDK4 and PCNA, on the other hand, overexpression of TMEM65 reduced the protein expression of tumor suppressive P53 and P21^cip1^ (Fig. 2F). Moreover, ectopic expression of TMEM65 inhibited cell apoptosis of AGS cells (*P* < 0.05; Fig. 2G). The TMEM65 induced decrease in apoptosis was confirmed by the active protein expression levels of key apoptosis markers (cleaved-caspase-3, cleaved-caspase-7, cleaved-caspase-8, cleaved-caspase-9 and cleaved-PARP) (Fig. 2H).

### TMEM65 promotes GC cells migration and invasion ability in vitro

To determine the effect of TMEM65 on migration and invasion abilities in GC, we performed wound healing assay and Matrigel invasion assay. Ectopic expression of TMEM65 markedly promoted cell migration ability in AGS cells. Quantitative analysis confirmed a significant increase in wound closure in TMEM65-expressing cells compared with control cells (*P* < 0.01; Fig. 2I). Matrigel invasion assay showed that TMEM65 also significantly promoted the invasiveness of AGS cells (*P* < 0.001; Fig. 2J). Western blot was then performed to verify the effect of TMEM65 on epithelial-mesenchymal transition (EMT) markers. As shown in Fig. 2K, ectopic expression of TMEM65 promoted EMT by increasing the expression of N-cadherin and β-catenin and decreasing the expression of E-cadherin. Therefore, these findings suggested that TMEM65 exerted its oncogenic effect by promoting cell migration and invasion in GC cells.

### TMEM65 knockdown suppresses GC cells viability, migration and invasion ability in vitro

**Fig. 3:**
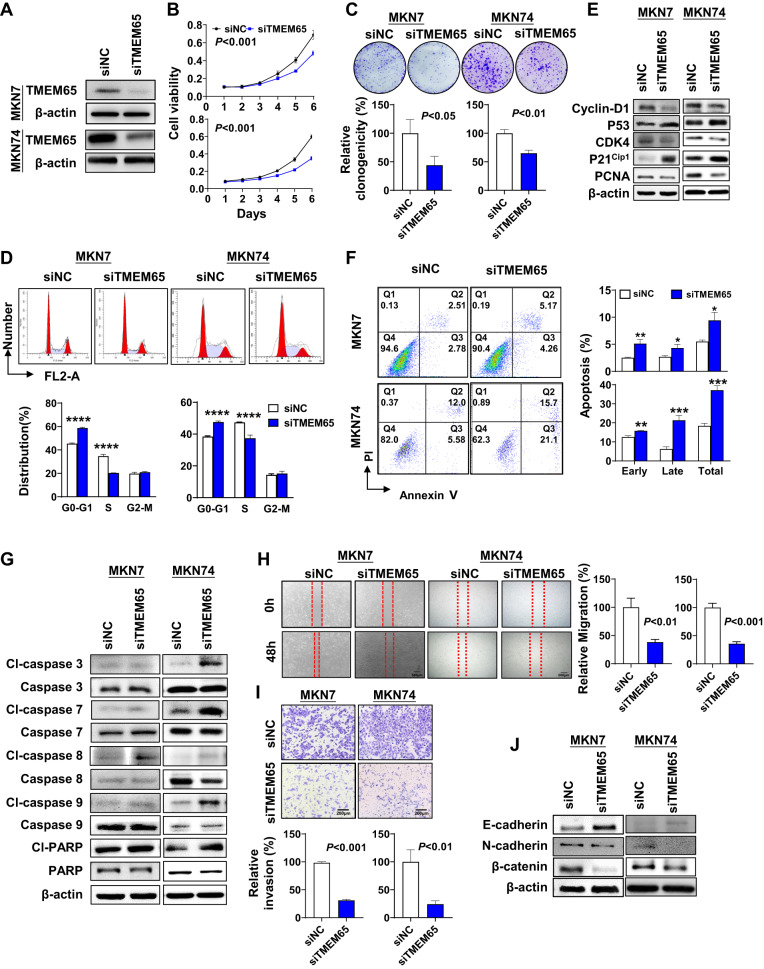
Knockdown of TMEM65 in GC cells suppresses cells growth, migration and invasion in vitro. **A** Knockdown of TMEM65 by siRNA in MKN7 and MKN74 was confirmed by Western blot. **B**, **C** Knockdown of TMEM65 in MKN7 and MKN74 cells significantly inhibited cell viability **B** and colony formation ability **C**. **D** Knockdown of TMEM65 in MKN7 and MKN74 cells caused G1-S arrest which was determined by flow cytometry. **E** Knockdown of TMEM65 decreased the protein expression of Cyclin-D1, CDK4, PCNA and increased the expression of P53, P21^cip1^. **F** Knockdown of TMEM65 in MKN7 and MKN74 cells promoted early and late apoptosis, determined by flow cytometry. **G** Western blot showed TMEM65 knockdown increased the expression level of cleaved-caspase-3, cleaved-caspase-7, cleaved-caspase-8, cleaved-caspase-9, and cleaved-PARP. **H** Knockdown of TMEM65 significantly inhibited migration ability in MKN7 and MKN74 cells. **I** Knockdown of TMEM65 significantly decreased cell invasion ability in MKN7 and MKN74 cells. **J** Western blot showed knockdown of TMEM65 decreased the protein expression of N-cadherin, β-catenin, and increased the expression of E-cadherin.

With the observation of ectopic expression of TMEM65 promoting GC cell growth, we further verified the effect of TMEM65 in GC using TMEM65 knockdown in GC cells (MKN7, MKN74) by siTMEM65. The knockdown efficiency was verified by Western blot (Fig. 3A). Knockdown of TMEM65 significantly inhibited cell viability in MKN7 and MKN74 cells (*P* < 0.001; Fig. 3B). Consistently, knockdown of TMEM65 inhibited colony formation ability in MKN7 and MKN74 (*P* < 0.05; Fig. 3C), caused G1-S arrest (*P* < 0.001; Fig. 3D), while promoted cell apoptosis in MKN7 and MKN74 cells (*P* < 0.05; Fig. 3F). The effects of TMEM65 knockdown in inducing cell cycle arrest was confirmed by Western blot for the protein expression of key cell cycle related markers including enhanced P53 and P21^cip1^ but reduced Cyclin-D1, CDK4 and PCNA (Fig. 3E). TMEM65 knockdown also promoted the protein expression of apoptosis related markers including cleaved-caspase-3, cleaved-caspase-7, cleaved-caspase-8, cleaved-caspase-9 and cleaved-PARP (Fig. 3G). Our results collectively indicated that TMEM65 promotes GC growth by promoting G1-S cell cycle progression and inhibiting cell apoptosis.

The wound healing assay and Matrigel invasion assay showed that knockdown of TMEM65 could significantly inhibited the migration ability and invasiveness of MKN7 and MKN74 cells (*P* < 0.01; Fig. 3H and I). Western blot of EMT markers also showed that knockdown of the TMEM65 caused the opposite result of overexpression (Fig. 3J).

### TMEM65 knockdown inhibited GC cells growth in vivo

**Fig. 4:**
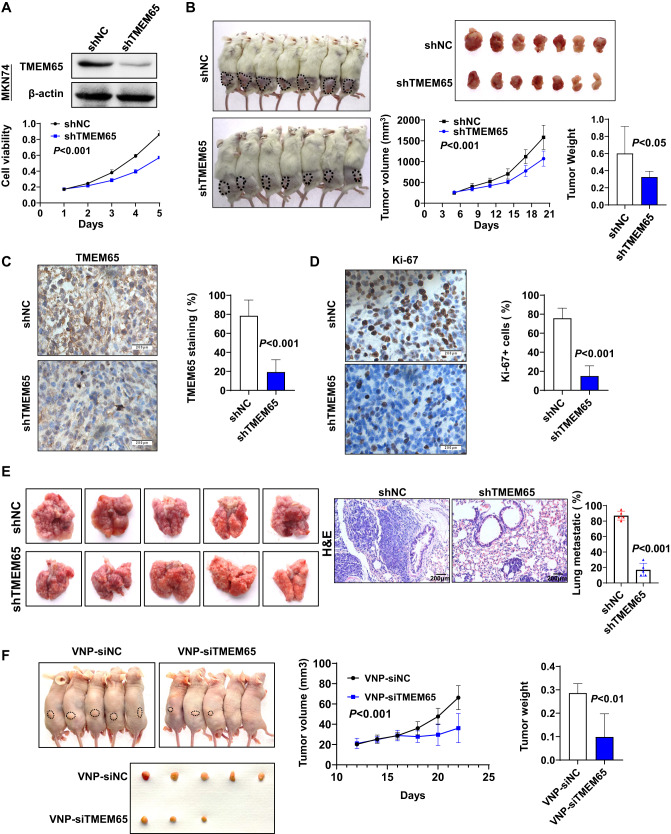
Knockdown of TMEM65 inhibits tumor growth and migration in vivo and TMEM65 is a therapeutic target in GC. **A** Knockdown of TMEM65 by shRNA in MKN74 was confirmed by Western blot, and stably knockdown of TMEM65 significantly inhibited cell viability. **B** Knockdown of TMEM65 in MKN74 cells significantly inhibited xenograft tumor growth and tumor weight of NOG mice, as shown by the growth curve of tumor volume at the experiment. **C** Knockdown efficiency of TMEM65 in xenografts was verified by IHC. **D** Knockdown of TMEM65 decreased the Ki67 positive cells percentage in subcutaneous xenografts, determined by IHC staining. **E** Knockdown of TMEM65 inhibited lung metastasis in nude mice. **F** Targeting depletion of TMEM65 by VNP-encapsulated TMEM65-siRNA significantly reduced tumor volume and tumor weight of nude mice, as shown by the growth curve of tumor volume and the tumor weight at the end of the experiment.

We further verified the tumor-promoting properties of TMEM65 in vivo. Stable transfection of shTMEM65 was used to establish knockdown MKN74 cell lines. The knockdown efficiency of TMEM65 was verified by Western blot (Fig. 4A), and knockdown of TMEM65 significantly inhibited cell viability (*P* < 0.001; Fig. 4A). We subcutaneously injected MKN74 cells stably knocked down TMEM65 (shTMEM65) or negative control (shNC) into the NOG mice, respectively. The results showed that TMEM65 knockdown significantly reduced tumor volume (*P* < 0.001, Fig. 4B) and tumor weight (*P* < 0.05, Fig. 4B). IHC of TMEM65 verified the knockdown efficiency (*P* < 0.001; Fig. 4C), and the Ki-67 positive cells by IHC confirmed that knockdown of TMEM65 inhibited cell proliferation, which was consistent with in vitro findings (*P* < 0.001; Fig. 4D).

### TMEM65 knockdown inhibits lung metastasis of GC cells in vivo

To verify the effect of TMEM65 on cell migration in vivo, we further assessed the effect of TMEM65 on tumor metastasis using shTMEM65 to establish stably knockdown TMEM65 in MKN74 cell lines. We subcutaneously injected MKN74 cells stably knocked down TMEM65 or negative control into the tail vein of nude mice, respectively. After four weeks, the mice were sacrificed. The area of lung metastatic under the same field was significantly decreased in TMEM65-knockdown group than in control group (*P* < 0.001; Fig. 4E). Overall, these results suggest that knockdown of TMEM65 inhibits metastasis of GC cells in vivo.

### Targeting TMEM65 by VNP-siTMEM65 significantly inhibits GC tumor growth in vivo

We further verified the therapeutic effect, in terms of targeting depletion of TMEM65 by VNP-encapsulated TMEM65-siRNA using subcutaneous xenograft mouse model. We subcutaneously injected wild type MKN74 cells (1 × 10^7^) into the nude mice in both experimental group or control group. We injected VNP-siTMEM65 or VNP-siNC into the xenograft tumor in the experimental group and the control group, the results showed that targeting depletion of TMEM65 by VNP-siTMEM65 in GC cells significantly reduced tumor volume (*P* < 0.001; Fig. 4F) and tumor weight (*P* < 0.01; Fig. 4F). Collectively, our results suggest that TMEM65 is a therapeutic target in GC.

### TMEM65 activates the PI3K/Akt/mTOR pathway

**Fig. 5:**
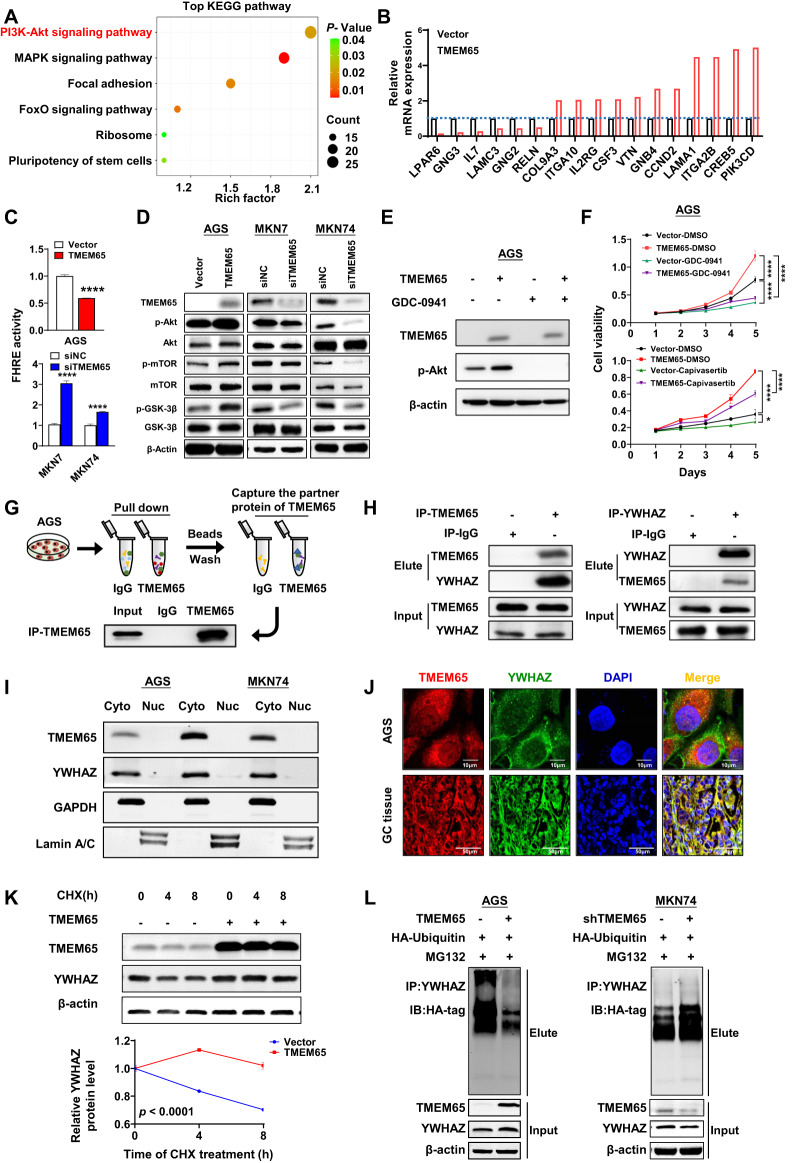
TMEM65 activates PI3K/Akt/mTOR signaling and YWHAZ is the downstream target of TMEM65. **A** RNA sequencing followed by KEGG pathways analysis revealed TMEM65 activated PI3K/Akt signaling. **B** Gene expression changes after overexpression of TMEM65 in PI3K/Akt signaling. **C** Ectopic expression of TMEM65 significantly reduced PI3K/Akt activity while knockdown of TMEM65 showed opposite effect. **D** Overexpression of TMEM65 enhanced the protein expression of PI3K/Akt/mTOR signaling related markers in GC cells, while knockdown of TMEM65 showed the opposite effect. **E** The inhibition efficiency on p-Akt of PI3K inhibitor GDC-0941 was confirmed by Western blot. **F** AGS cells treatment with PI3K inhibitor GDC-0941 (0.5 μmol/L) and Capivasertib (1 nmol/L), inhibited the promoting effect of TMEM65 on cell viability. **G** Workflow of downstream factor identification of TMEM65 by LC-MS. **H** Co-IP followed by Western blot analyses confirmed the binding between TMEM65 and YWHAZ in AGS cells. **I** TMEM65 and YWHAZ co-localized in cytoplasm of GC cells (AGS, MKN74 and MKN7) determined by Western blot. **J** TMEM65 and YWHAZ are mainly co-localized in cytoplasm in AGS and GC tissues as demonstrated by fluorescence microscope. **K** Ectopic expression of TMEM65 increased the stability of YWHAZ in AGS cells. **L** TMEM65 decreased YWHAZ ubiquitination and increased its protein level.

To further understand the molecular mechanism of TMEM65, gene expression profiles were analyzed by RNA-sequencing in TMEM65-overexpressing cells compared with vector-transfected counterparts. KEGG pathway enrichment analysis was performed on the differentially expressed genes. The results showed that TMEM65 mainly induced the oncogenic PI3K/Akt and MAPK signaling pathways (Fig. 5A). PI3K/Akt signaling, with the most genes differentially expressed by TMEM65, is of most interest. Gene expression changes after overexpression of TMEM65 in PI3K/Akt signaling was analyzed by RNA-sequencing, including LPAR6, GNG3, IL7 and other genes. (Fig. 5B). The relationship between TMEM65 and 5 of these genes (IL7, ITGA2B, LAMA1, COL9A3, VTN) was showed in Supplementary material Fig. S1. We further performed luciferase experiments of PI3K/Akt (FHRE-Luc) in TMEM65 overexpression and knockdown GC cell lines as compared with control cells. The results showed that overexpression of TMEM65 in AGS cells would inhibit the activity of FHRE-Luc (*P* < 0.0001; Fig. 5C), accompanied with increased protein levels of phospho-Akt (p-Akt), phospho-glycogen synthase kinase-3beta (p-GSK3β) and phospho-mammalian target of rapamycin (p-mTOR) as shown by Western blot (Fig. 5D). On the contrary, knockdown of TMEM65 displayed the opposite result (Fig. 5C and D). To further verify the effect of TMEM65 on the PI3K/Akt signaling, PI3K inhibiter GDC-0941 or Akt inhibiter Capivasertib were used [10–12]. GDC-0941 decrease the p-Akt increase effect by TMEM65 ectopic expression as compared with DMSO treatment (Fig. 5E). Consistently, cell viability assays showed that GDC-0941 or Capivasertib reduced the promoting effect of overexpression of TMEM65 (*P* < 0.05; Fig. 5F). Thus, our results indicated that TMEM65 exerts its oncogenic effect at least in part by activating the oncogenic PI3K/Akt/mTOR pathway.

### TMEM65 interacts with YWHAZ in GC cells

To gain insight into the molecular mechanistic basis of the tumor promoting effect of TMEM65 in GC, we sought to identify its interacting partners by Co-IP followed by identification of the associated proteins using LC-MS (Fig. 5G). Among the identified 208 candidates (Table S2), 31 were cytosolic proteins and related to PI3K/Akt signaling pathway, and 10 candidates were overexpressed in GC (YWHAZ, LDHA, ENO1, IQGAP1, ALDOA, HSP90AB1, VDAC1, EEF2, ATP4A, HSP90AA1) [13–22]. We next selected YWHAZ for further evaluation in light of its reported roles in cancer [13, 23, 24]. To validate the interaction between TMEM65 and YWHAZ were co-expressed in GC cells, and total proteins were immunoprecipitated with anti-TMEM65 or anti-YWHAZ antibodies. Western blot analyses confirmed that TMEM65 and YWHAZ could be coprecipitated by each other in GC cells (Fig. 5H). Then we extracted the cytoplasm and nucleus protein of GC cells of AGS, MKN7 and MKN74, the result shows that TMEM65 and YWHAZ were mainly localized in the cytoplasm in GC cells (Fig. 5I). We further examined the intracellular distribution of TMEM65 and YWHAZ by immunofluorescence staining. Confocal microscopy images showed that TMEM65 and YWHAZ were co-localized in the cytoplasm in TMEM65 overexpressed AGS cells and GC tissues (Fig. 5J). We further determined whether TMEM65 regulated protein stability of YWHAZ. We treated TMEM65 or control vector transfected cells with the protein synthesis inhibitor cycloheximide (CHX). As shown in Fig. 5K, YWHAZ was stabilized in the presence of TMEM65 in AGS cells (*P* < 0.0001). We next investigated whether TMEM65 increased the protein stability of YWHAZ by regulating its ubiquitination and degradation. We transfected TMEM65 overexpressed or knockdown cells with ubiquitin-HA, followed by MG132 treatment and pulldown of cell lysates with anti-YWHAZ. As shown in Fig. 5L, TMEM65 overexpression decreased YWHAZ ubiquitination, while TMEM65 knockdown had an opposite effect, suggesting that TMEM65 binds YWHAZ to inhibit ubiquitin-mediated degradation of YWHAZ.

### Oncogenic function of TMEM65 depends on YWHAZ

**Fig. 6:**
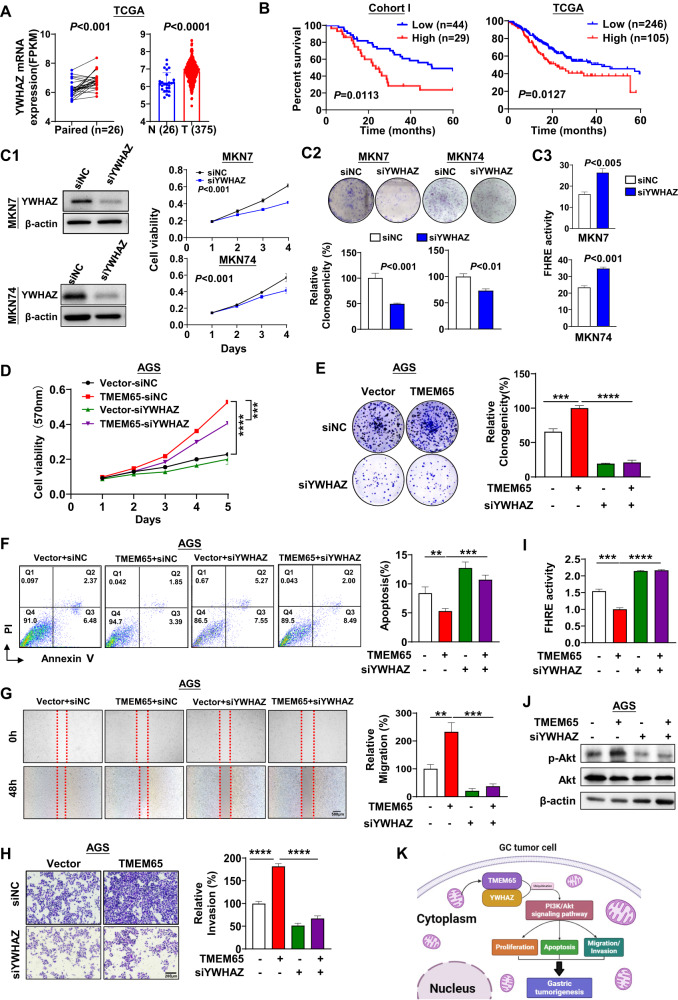
Oncogenic function of TMEM65 is depended on YWHAZ in GC cells. **A** YWHAZ was upregulated in GC tissues as compared with normal tissues from TCGA cohort. **B** Kaplan-Meier survival analysis from cohort I and TCGA cohort showed GC patients with high YWHAZ expression had poorer survival. **C1** Knockdown of YWHAZ in MKN7 and MKN74 cells was confirmed by Western blot. **C1–C3** Knockdown of YWHAZ in MKN7 and MKN74 cells significantly inhibited cell viability **C1**, colony formation ability **C2** and PI3K/Akt signaling, evidenced by increased FHRE activity **C3**. **D**–**H** Knockdown of YWHAZ in AGS cells significantly reduced the promoting effect of TMEM65 on cell viability **D**, cell clonogenicity **E**, anti-apoptosis effect **F**, cell migration ability **G**, cell invasiveness **H**, and activation of PI3K/Akt signaling, determined by luciferase reporter assay **I** and Western blot **J**. **K** Schematic illustration of the molecular mechanism of TMEM65 in GC.

We investigated the clinical significance of YWHAZ in GC. YWHAZ is overexpressed in paired GC tissues compared to adjacent normal tissues from TCGA cohort (*n* = 26; *P* < 0.01), as well as in the overall cohort (adjacent normal, *n* = 26; GC, *n* = 375) (Fig. 6A). GC patients with high YWHAZ expression also showed poor survival from cohort I and TCGA cohort (*P* < 0.05; Fig. 6B). We determined expression of YWHAZ in AGS, MKN7 and MKN74 cells, and revealed that MKN74 and MKN7 cells had higher YWHAZ expression compared to AGS cells (Fig. S4). We thus depleted YWHAZ in MKN7 and MKN74 cells by siRNA (Fig. 6C1). YWHAZ knockdown significantly decreased cell growth and colony formation (*P* < 0.01) (Fig. 6C1–2) and promoted FHRE-Luc, indicative of suppressed PI3K/Akt signaling (*P* < 0.005) (Fig. 6C3). We then performed knockdown of YWHAZ in TMEM65 overexpressing AGS cells. YWHAZ knockdown blocked the promoting effect of TMEM65 overexpression on cell viability, colony formation, apoptosis and cell migration/invasiveness (*P* < 0.01; Fig. 6D–H). Moreover, knockdown of YWHAZ blunted the TMEM65 induced PI3K/Akt pathway activation (*P* < 0.001; Fig. 6I–J).

## Discussion

In this study, TMEM65 amplification was identified by genomic hybridization microarray to profile copy-number variations in GC. We revealed that the amplification of TMEM65 was positively associated with its mRNA overexpression in 413 GC cases in TCGA cohort (*P* < 0.001), suggesting that increased TMEM65 copy number contributes to the upregulation of TMEM65. TMEM65 was located on chromosome 8q24. which is one of the most frequently amplified regions in multiple cancers [4, 25, 26]. We investigated the clinical importance of TMEM65 expression in GC patients at mRNA and protein levels in two independent cohorts. Patients with high TMEM65 expression showed lower survival rate (All stage and Stage III-IV). Both univariate and multivariate Cox regression analyses showed high expression levels of TMEM65 were correlated with an increased risk of GC-related death. Consistently, TMEM65 copy number gain was significantly correlated with the poor survival and was an independent prognostic factor for GC patients from our cohort (*P* = 0.0042). The clinical outcome of GC varies greatly depending on the aggressiveness of individual tumors. Many patients experience disease recurrence following radical surgery. Thus, additional prognostic biomarkers may provide better risk assessment that can guide personalized chemotherapy. Our results demonstrated that TMEM65 may serve as a new independent prognostic marker for patients with GC.

Since TMEM65 was commonly upregulated in patients with GC, this implies its important oncogenic function during gastric tumorigenesis. With this connection, we performed in vitro gain- and loss-of-function assays on TMEM65. In vitro study demonstrated that overexpression of TMEM65 in AGS cells significantly promoted cell proliferation. Conversely, knockdown of TMEM65 in MKN7 and MKN74 cells significantly decreased cell growth. TMEM65 promoted GC cell growth was mediated by promoting G1-S cell cycle transition and inhibiting cell apoptosis. TMEM65 reduced the expression of P53, P21^cip1^, P53 as a transcription factor induces the expression of P21^cip1^, which is an inhibitor of the cyclin dependent kinases (CDKs) [27]. Loss the inhibition effect of P21^cip1^, TMEM65 thus promoted G1-S transition, associated with the upregulation of cyclin D1 and CDK4, which are the key regulator of the transition through the G1 phase of the cell cycle. Concomitantly, the growth-enhancement effect of TMEM65 was related to inhibition of apoptosis. Overexpression of TMEM65 inhibited caspase-dependent apoptosis pathway including caspase-3, caspase-7, caspase-8, caspase-9, and PARP. Caspase dependent apoptosis involved a group of intracellular proteases called caspases are responsible for the deliberate disassembly of the cell into apoptotic bodies during apoptosis [28, 29]. Caspases are present as inactive pro-enzymes that are activated by proteolytic cleavage. Caspases 8, 9 and 3 are situated at pivotal junctions in apoptosis pathways. Moreover, ectopic expression of TMEM65 in GC cell lines significantly promoted their migration and invasion abilities. Notably, knockdown of TMEM65 in MKN74 cells inhibited cell proliferation in a subcutaneous xenograft model and lung metastasis model. Collectively, our results indicate that TMEM65 exerts oncogenic properties in GC via promoting cell proliferation and cell-cycle progression, inhibiting apoptosis and increasing metastatic abilities.

Given the important role of TMEM65 in GC tumorigenesis and metastasis, we evaluated its potential as a therapeutic target. In line with the oncogenic function of TMEM65, targeting depletion of TMEM65 by VNP-encapsulated TMEM65-siRNA significantly suppressed tumor growth in subcutaneous xenograft tumor model, and TMEM65 could serve as a therapeutic target in GC. Taken together, our results suggest that targeting of TMEM65 is a promising strategy for the treatment of GC patients.

We demonstrated that TMEM65 plays an oncogenic role via activating PI3K/Akt/mTOR signaling pathway in GC cells, which identified by RNA sequencing analysis [30–32]. As we all know, PI3K/Akt/mTOR signaling is one of the most frequently dysregulated signaling cascades in human malignancies and is implicated in a wide variety of different neoplasms [30–33]. More and more evidence showed that the PI3K/Akt/mTOR pathway is one of the crucial factors for GC cells proliferation and survival, and can promote the EMT transformation of GC cells [34, 35]. Ectopic expression of TMEM65 caused significant induction and activation of PI3K/Akt/mTOR and induced phosphorylation of Akt, mTOR and GSK-3β. Furthermore, PI3K/Akt signaling inhibitor abolished the oncogenic effect of TMEM65 on cell proliferation. Thus, these results suggest that TMEM65 plays an oncogenic role mainly through activation of PI3K/Akt/mTOR signaling pathway.

To understand the molecular basis of the role of TMEM65, Co-IP of TMEM65 followed by LC-MS identified YWHAZ as an interacting partner of TMEM65. The direct interaction between TMEM65 and YWHAZ was confirmed by Western blot analysis of Co-IP products. TMEM65 was co-localized in the cytoplasm with YWHAZ by confocal immunofluorescence assay and Western blot analysis. And the mRNA expression of TMEM65 and YWHAZ were positively correlated in TCGA cohort and cohort I. These results suggested that YWHAZ is a direct downstream interaction partner of TMEM65. Moreover, we demonstrated that TMEM65 interacts with YWHAZ and stabilized the latter against ubiquitination-mediated degradation. Notably, YWHAZ plays a pivotal role in TMEM65-mediated activation of PI3K/Akt/mTOR signaling in gastric tumorigenesis [36, 37]. YWHAZ belongs to the 14.3.3 protein family which mediates signal transduction by binding to phosphoserine-containing proteins. YWHAZ has been reported to promote tumor growth in several cancer types and involved in PI3K/Akt signaling [38, 39], emphasized YWHAZ as a prognostic factor and potential therapeutic target for GC [24]. In keeping with this, we revealed that expression of YWHAZ significantly enhanced in GC, signifying its oncogenic role in GC. We then demonstrated that overexpressed TMEM65 could stabilized YWHAZ protein to facilitate its oncogenic function in GC. TMEM65 promoted GC growth, invasion/migration ability and anti-apoptosis through coordinating with a novel interacting partner YWHAZ. We demonstrated that YWHAZ knockdown in GC cells blunted the TMEM65-induced oncogenic function and PI3K/Akt signaling activation [40], inferring that the oncogenic role of TMEM65 in GC is, at least in part, dependent on YWHAZ.

In conclusion, we demonstrate that TMEM65 is amplification in GC due to copy number gain. High TMEM65 expression or copy number gain are associated with poor survival of GC patients. TMEM65 is a novel GC oncogenic factor that promotes cell proliferation, cell cycle transition, metastasis but inhibits cell apoptosis. Mechanistically, TMEM65 binds and stabilized YWHAZ to activate PI3K/Akt/mTOR signaling pathway to facilitate GC. Targeting of TMEM65 is a promising strategy for treatment of GC patients (Fig. 6K).

## Materials and methods

### Human gastric tissue collection

We recruited 207 GC patients including cohort I of 78 GC tumor tissues, and Cohort II of 129 GC tumor tissues from Peking University Cancer Hospital, Beijing, China. The clinical and pathological characteristics of patients in cohort I were listed in Table S1. We also included TCGA cohort of 375 GC samples in this study. In addition, 21 paired paraffin-embedded slides from primary gastric tumor and adjacent nontumor sites from patients with GC were obtained at the First Hospital of Hebei Medical University, Shijiazhuang, China. The specimens were snap-frozen in liquid nitrogen and stored at −80 °Cor fixed in 10% formalin and embedded in paraffin for routine histologic examination. The study protocol was approved by the Clinical Research Ethics Committee of The First Hospital of Hebei Medical University and Peking University Cancer Hospital and Institute. And all subjects provided informed consent for obtaining the study specimens. This study was carried out in accordance with the Declaration of Helsinki of the World Medical Association.

### GC cell lines

GC cell lines (MKN74, AGS, MKN7) were used in this study. AGS were obtained from ATCC (American Type Culture Collection, Manassas, VA). MKN74 was obtained from JCRB (Japanese Collection of Research Bioresources Cell Bank, Japan). MKN7 was obtained from BNCC (BeNa Culture Collection, China). AGS and MKN74 were obtained between 2014 and 2015 and MKN7 were obtained 2023. These cells authentication was confirmed by short tandem repeat profiling. Cells were cultured and maintained in Dulbecco’s Modified Eagle’s Medium (DMEM, Gibco BRL) supplemented with 10% fetal bovine serum (FBS, Gibco BRL) and 1% antibiotics according to the ATCC protocols. Cells were maintained at a 37 °C incubator with 5% CO2. Routine Mycoplasma testing was performed by PCR. Cells were grown for no more than 25 passages in total for any experiment.

### RNA extraction, semi-quantitative RT-PCR, and real-time PCR analyses

Total RNA was extracted from cells and tissues using TRIzol Reagent (Invitrogen). The extracted RNA was reversely transcribed into complementary DNA (cDNA) through a cDNA Reverse Transcription Kit (TransGen Biotech). Semi-quantitative PCR was performed by AmpliTaq Gold DNA polymerase (Applied Biosystems; Thermo Fisher Scientific). Quantitative real-time PCR was performed by SYBR Green PCR Master Mix (Takara) on 7500HT Fast Real-Time PCR System (Applied Biosystems; Thermo Fisher Scientific). Experiments were repeated twice. β-actin was tested for normalization. Each sample was tested in triplicate. The 2−ΔΔCt method was employed to quantify the relative gene expression levels. The sequences of primers used are listed in Supplementary Table S3.

### RNA interference

TMEM65 siRNA (siTMEM65: 5’-CAGGACAGCUGAGAUAUGUTT-3’), TMEM65 shRNA (shTMEM65: 5’-CAGGACAGCUGAGAUAUGUTT-3’), YWHAZ siRNA (siYWHAZ: 5’-CACGCUAAUAAUGCAAUUATT-3’) and Negative Control (siNC) or (shNC) were ordered form Genepharma Company. 50 nmol of siTMEM65, shTMEM65, siYWHAZ or siNC, shNC were transfected into cells using Lipofectamine 2000 (Invitrogen) according to the manufacturer’s instructions.

### Subcutaneous xenograft and lung metastasis mouse models

MKN74 cells were stably transfected with shTMEM65 or sh-negative control (shNC), after transfection, MKN74 cells were selected by puromycin. The effect of shTMEM65 stable cells was verified by Western blot and cell viability assay. According to the random number table, 14 male NOG mice were divided into the control group (*n* = 7), and experiment group (*n* = 7). MKN74(1 × 10^7^ cells) were injected subcutaneously into the right flanks of 4-week-old male NOG mice. Tumor volumes were measured every 3 days using a caliper. Tumor volume (mm^3^) was estimated by measuring the longest and shortest diameters of the tumor and calculated as previously described. Two weeks later, mice were sacrificed. At the endpoint, tumors were harvested and weighted. The excised tissues were either fixed in 10% neutral-buffered formalin. Tumor sections from paraffin-embedded blocks were used for histologic examination.

To assess the effect of TMEM65 on tumor metastasis, MKN74 cells (5 × 10^6^ cells) stably transfected with shTMEM65 or shNC were injected into the tail vein of 6-week-old male Balb/c nude mice (*n* = 5 per group). Four weeks after injection, the mice were sacrificed. The lungs were stained with hematoxylin and eosin. The area of lung metastatic under the same field were counted in a blinded manner. All experimental procedures were approved by the Animal Ethics Committee of the Chinese University of Hong Kong.

### RNA sequencing and analysis

About 1 × 10^7^ cells per sample was used as input material for the RNA sample preparations. All samples had RNA integrity values > 6.8. Sequencing libraries were generated using the IlluminaTruSeq RNA Sample Preparation Kit (Illumina) following the manufacturer’s recommendations. The libraries were sequenced using the Illumina HiSeq X-ten platform as per the manufacturer’s instructions (Shanghai Biotechnology Corp, Shanghai, China). Gene expression profiles were analyzed in TMEM65-overexpressing cells compared with control vector-transfected counterparts. KEGG pathway enrichment analysis was conducted using the differential genes of the overexpressed TMEM65 group and the control group, and the top 6 pathways were sorted out according to the number of enriched differential genes and *P* value.

### Co-immunoprecipitation (Co-IP) and liquid chromatography-mass spectrometry

Briefly, total protein was extracted in radioimmunoprecipitation assay (RIPA) buffer supplemented with proteinase inhibitor and DNase I (GD201-01, TransGen Biotech). Immunoprecipitation was performed using anti-TMEM65 antibody (ab236861, Abcam) or anti-IgG Antibody. Antibody and lysed protein were stirred overnight at 4 °C. We use Pure Proteome™ Protein A/G Mix magnetic beads (88802, Thermo Scientific™) to specifically bind the protein of interest, the magnetic beads were collected by hand on a magnetic stand. Collected beads and used Low-pH Elution Buffer (0.1 M glycine, pH2.0) to elute the target protein and neutralize the PH. Beads with extracted proteins were washed 3 times by 50 mM ammonium bicarbonate buffer and subjected to digestion by trypsin at 37 °C for 2 h (Promega, Madison, WI). The antibodies used are listed in Table S4. Tryptic peptides were then extracted for liquid chromatography-mass spectrometry (LC-MS) analysis (Shanghai Biotechnology Corp, Shanghai, China) [41]. The candidate TMEM65 interacts proteins identified by LC-MS were shown in Table S2.

### Ubiquitination assay

TMEM65 overexpressed AGS cells or TMEM65 knockdown MKN74 cells were co-transfected with Ubiquitin-HA for 24 h. Then the cells were incubated in the presence or absence of 10 µM MG132 (HY-13259C, MedChemExpress) for 12 h. Cells were lysed with RIPA buffer supplemented with protease inhibitors. Immunoprecipitation was performed using anti-YWHAZ (14881-1-AP, Proteintech). Immunoprecipitated proteins were analyzed by Western blot using anti-HA (51064-2-AP, Proteintech).

### Immunofluorescence

AGS cells (TMEM65 overexpressed) were seeded onto coverslips in a 6-well plate. 24 h after transfection, cells were fixed with 4% paraformaldehyde and permeabilized with 0.2% Triton X-100, blocked in Normal Goat Serum, and incubated with anti-TMEM65 (1:100 dilution) and anti-YWHAZ (1:200 dilution) overnight at 4 °C, followed by anti-Rabbit IgG secondary fluorescent antibody and anti-mouse IgG fluorescent secondary antibody in the dark for 1 h. Cells were then mounted with ProLong Gold Antifade Mountant with DAPI (Life Technologies). Pictures were taken with a fluorescence microscope, and the slides were wrapped in tin foil and stored at −20 °C.

### Vesicle-like PLGA-based nanoparticle (VNP) formulation

Vesicle-like PLGA-based nanoparticle (VNP) formulation Vesicle-like PLGA-based nanoparticle were assembled by Guangzhou Kelan Biotechnology Co., Ltd (Guangzhou, China). 2’-O-Methyl (2’-OMe) modified siRNA with 2’-O-Methyl (2’-OMe) modification were purchased from GenePharma Co.,Ltd (Shanghai, China). The sequencing of human siTMEM65 (sense: GUGGCAAACACGUCUUAGUTT; antisense: ACUAAGACGUGUUUGCCACTT. We subcutaneously injected wild type MKN74 cells (1 × 10^7^) into the nude mice of 4-week-old male Balb/c nude mice (*n* = 5 per group). On day 16, the experimental and control groups were injected with VNP-siTMEM65 or VNP-siNC once every two days in xenograft tumors, tumor volumes were measured every 2 days using a caliper. Tumor volume (mm^3^) was estimated by measuring the longest and shortest diameters of the tumor and calculated as previously described. One month later, mice were sacrificed. At the endpoint, tumors were harvested and weighted. We weighed the transplanted tumor in the two group.

### Statistical analysis

The results were expressed as mean ± SD. The Pearson correlation coefficient was used to evaluate the correlation between TMEM65 gene amplification and expression in the clinical samples. Overall survival in relation to expression was evaluated by the Kaplan-Meier survival curve and the log-rank test. Mann-Whitney *U* test or Student’s *t* test was performed to compare the variables of two groups. mRNA expression of 375 GC samples were downloaded from TCGA via UCSC Xena. The difference in cell viability and tumor growth rate between two groups of mice was determined by repeated-measures analysis of variance. In vitro experiments were performed in triplicates, with at least three independent experiments. All statistical tests were performed using Graphpad Prism 9.0 (Graphpad Software Inc., San Diego, CA) or the SPSS program (version 17.0; SPSS, Chicago, IL). *P* values < 0.05 were taken as statistical significance.

### Supplementary information

Supplementary Figures

Supplementary Tables

Supplementary method

## Data Availability

All datasets and raw data generated and/or analysed during the current study are available from the corresponding author upon reasonable request. The RNA-seq data are deposited with the GEO with accession code GSE254012.
