# Supplementary material for: TMEM65 promotes gastric tumorigenesis by targeting YWHAZ to activate PI3K-Akt-mTOR pathway and is a therapeutic target
Source: Oncogene. 2024 Feb 10;43(13):931–43. doi: 10.1038/s41388-024-02959-9 (PMC10959749; doi:10.1038/s41388-024-02959-9)
Supplement: Supplementary file 1 — Supplementary Figures [file 41388_2024_2959_MOESM1_ESM.pdf]

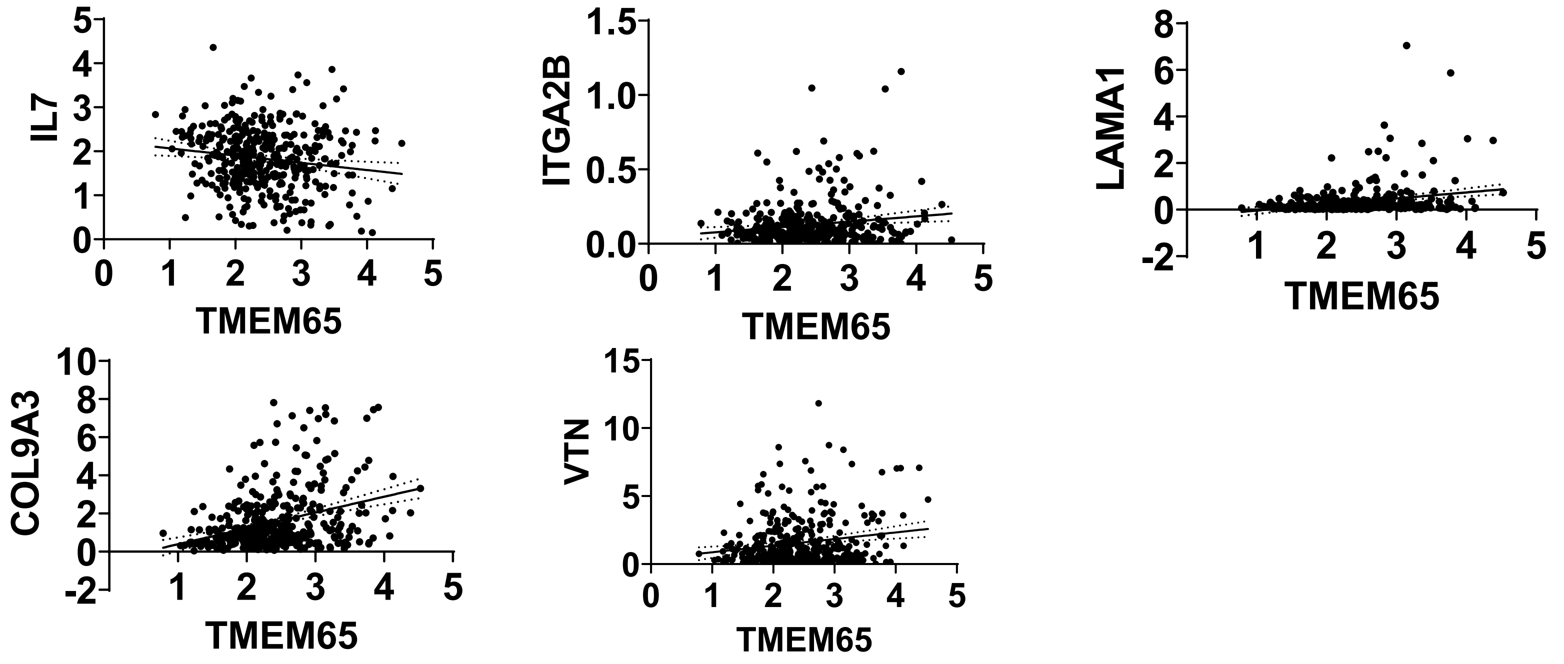

Figure S1. The relationship between TMEM65 and 5 expression changed genes after TMEM65 overexpressed.

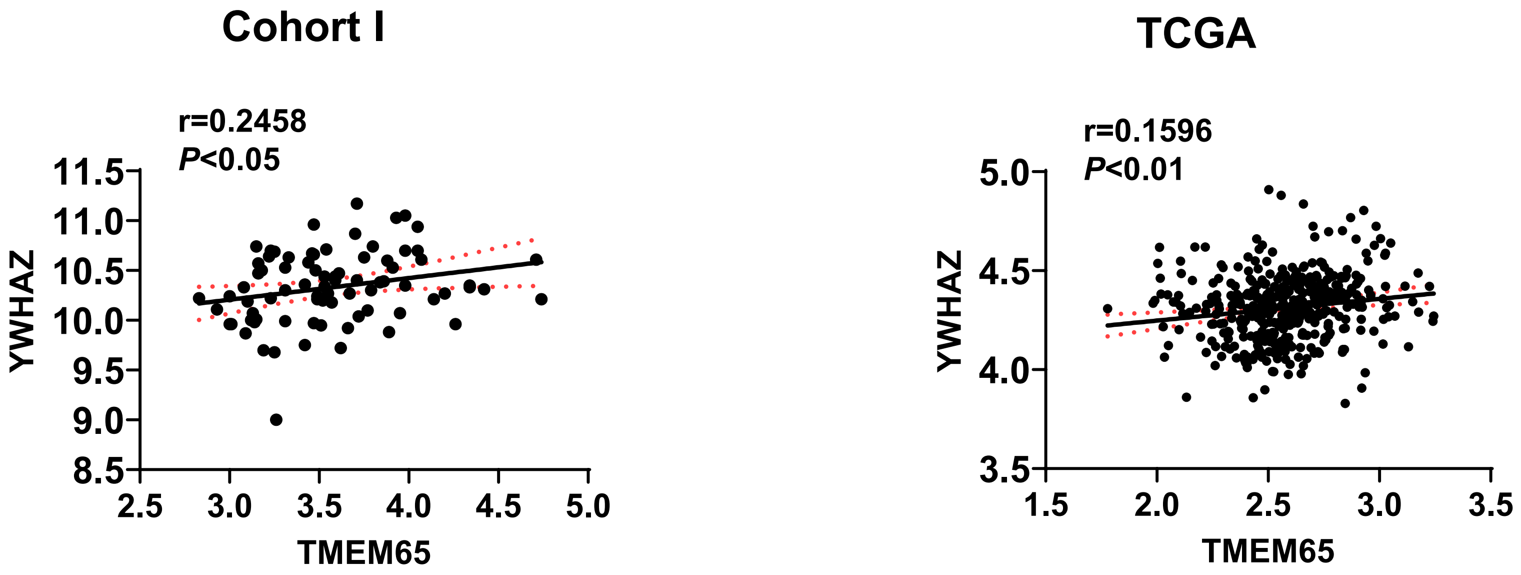

Figure S2. The relationship between TMEM65 and YWHAZ in cohort I and TCGA cohort.

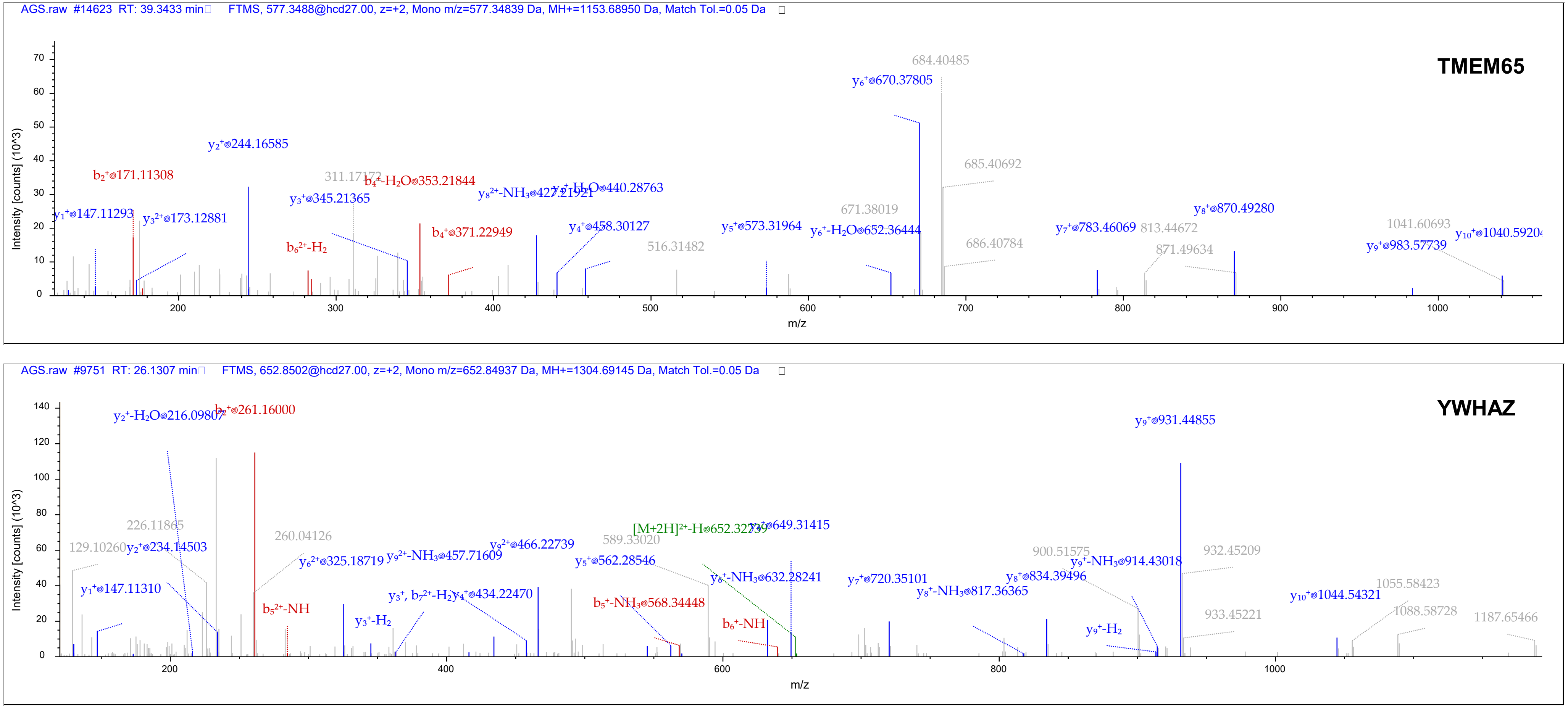

Figure S3. Liquid chromatography-mass spectrometry (LC-MS) of TMEM65 and YWHAZ.

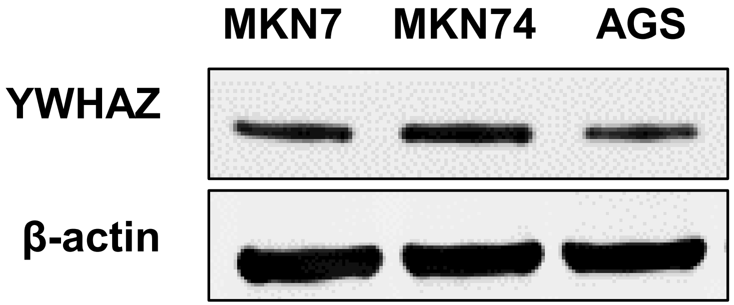

Figure S4. The basic expression of YWHAZ in AGS, MKN7 and MKN74.
