# Supplementary material for: TMEM65 promotes gastric tumorigenesis by targeting YWHAZ to activate PI3K-Akt-mTOR pathway and is a therapeutic target
Source: Oncogene. 2024 Feb 10;43(13):931–43. doi: 10.1038/s41388-024-02959-9 (PMC10959749; doi:10.1038/s41388-024-02959-9)
Supplement: Supplementary file 2 — Supplementary Tables [file 41388_2024_2959_MOESM2_ESM.docx]

**Table S1.** Clinical and pathological characteristics of patients in cohort Ⅰ.

|  | **Cohort I**  **(Beijing)** |
| --- | --- |
| **No. of patient** | 78 |
| **Age at resection** |  |
| <60 | 30 (38.5%) |
| >=60 | 48(61.5%) |
| **Gender** |  |
| Male | 58 (74.4%) |
| Female | 20 (25.6%) |
| **Location** |  |
| Cardia | 27(34.6%) |
| non-cardia | 51(65.4%) |
| **Differentiation** |  |
| Low | 15(19.2%) |
| High and moderate | 63(80.8%) |
| **Vascular cancer thrombus** |  |
| Positive | 36(46.1%) |
| Negative | 42(53.9%) |
| **T Stage** |  |
| I and II | 7(9.0%) |
| III and IV | 71(91.0%) |
| **N Stage** |  |
| Positive | 64(82.1%) |
| Negative | 14(17.9%) |
| **M Stage** |  |
| Positive | 10(12.8%) |
| Negative | 68(87.2%) |
| **TNM Stage** |  |
| I+ II | 20 (25.6%) |
| III+IV | 58 (74.4%) |
| **Survival** |  |
| 0: Live | 37 (47.4%) |
| 1: Dead | 41 (52.6%) |

**Table S2.** TMEM65-related proteins by co-IP pulled down in AGS.

| YWHAB | HNRNPD | CLTC | SF3B4 | SLC25A6 | ECPAS |
| --- | --- | --- | --- | --- | --- |
| YWHAH | HNRNPK | CLU | SFPQ | ALB | PDIA3 |
| YWHAQ | HNRNPM | CFL1 | HSPA9 | ADH1B | P4HB |
| YWHAZ | HNRNPU | CCDC141 | STRIP2 | SERPINF2 | FAM81A |
| PSMD2 | HNRNPA2B1 | CFI | TAF15 | ACTN4 | MAL2 |
| RPS12 | H1-2 | MACROH2A1 | CCT8 | ENO1 | S100A11 |
| RPS13 | H1-3 | CORO1A | TXN | ANXA1 | PTMA |
| RPS14 | H1-4 | DCD | TALDO1 | ANXA2 | PKM |
| RPS19 | H1-5 | DSP | TRIM28 | APOC3 | GDI2 |
| RPS23 | H2AX | MCM3 | TAGLN2 | RARS1 | IQGAP1 |
| RPS3 | H2BC1 | MCM4 | VCP | ATP5F1A | RTN4 |
| FAU | H3C1 | DNAJC2 | TKT | BZW1 | ARHGDIA |
| RPS5 | H3C15 | DAD1 | TMEM65 | ACTBL2 | RRBP1 |
| RPS6 | H4C1 | ETFA | TPI1 | MTHFD1 | ATP2A2 |
| RPS7 | LDHA | EEF1A2 | PRSS3 | RCAN3 | SAFB |
| RPS8 | LDHB | EEF1G | TUBA1B | CALM2 | SRSF8 |
| RPSA | LYZ | EEF2 | TUBB | CALML5 | PPP1CB |
| ALDH9A1 | MVP | TUFM | TUBB4B | CPS1 | TF |
| XRN2 | MDH1 | HSPA5 | RPS27A | CLIC1 | SNRPD2 |
| HSPD1 | MDH2 | HSP90B1 | UBA1 | CFAP45 | SLC12A2 |
| RPLP0 | MAP4 | EEF1E1 | VIL1 | ACTR3 | RAN |
| RPLP1 | MAPRE1 | EIF2S3 | VIM | AHCY | HSPA8 |
| RPL12 | TOMM22 | EIF3M | VCL | PHB | HSPH1 |
| RPL13 | PAICS | EIF5A | VDAC1 | PA2G4 | HSPB1 |
| RPL13A | MYL6 | EXOSC3 | XRCC5 | PLEC | HSP90AA1 |
| RPL14 | NAPSA | EZR | YBX1 | PCBP3 | HSP90AB1 |
| RPL17 | NACA | SSRP1 | YBX3 | PABPN1 |  |
| RPL18 | SNU13 | CAPZA2 | RPL3 | ASXL1 |  |
| RPL18A | NPC2 | FLNA | RPL30 | PTBP3 |  |
| RPL19 | NCL | ALDOA | RPL36 | ATP4A |  |
| RPL23A | NAP1L1 | ALDOC | RPL7A | LMNA |  |
| RPL27 | NAP1L4 | LGALS3BP | ACTA2 | GYG1 |  |
| RPL27A | PPIB | PRKCSH | ACTB | HBA1 |  |
| RPL28 | SLC25A3 | GAPDH | ACTG1 | HBB |  |

**Table S3.** DNA sequences of primers used in this study.

| **Primer name** | **Sequence (5'-3')** |
| --- | --- |
| β-actin-F | CATCCACGAAACTACCTTCAACTCC |
| β-actin-R | GAGCCGCCGATCCACACG |
| TMEM65-F | TGTTGCTGGAACCCATATTGAAA |
| TMEM65-R | CGTAGCCTGCAAGTCCAAGT |
| YWHAZ-F | ATGTACTTGGAAAAAGGCCG |
| YWHAZ-R | CCCTGCTCTTGAGGAGCTTA |

**Table S4.** Antibodies used in this study.

| **Antibody name** | **Company** | **Catalog No.** | **Dilution** |
| --- | --- | --- | --- |
| TMEM65 | Abcam | ab236861 | 1:1500 (WB), 1:200 (IHC), 1:100 (IF) |
| YWHAZ | Novus | NB100-1964 | 1:200 (IF) |
| YWHAZ | Santa | sc-518031 | 1:1000 (WB) |
| YWHAZ | Proteintech | 14881-1-AP | 10μg(IP) |
| HA | Proteintech | 51064-2-AP | 1:1000 (WB) |
| β-actin | Cell Signaling Technology | #4970 | 1:1000 (WB) |
| Cyclin-D1 | Cell Signaling Technology | #2922 | 1:1000 (WB) |
| P53 | Cell Signaling Technology | #2524 | 1:1000 (WB) |
| CDK4 | Cell Signaling Technology | #12790 | 1:1000 (WB) |
| P21cip1 | Cell Signaling Technology | #2947 | 1:1000 (WB) |
| PCNA | Cell Signaling Technology | #13110 | 1:1000 (WB) |
| \| E-cadherin \|  \|  \|  \| \| --- \| --- \| --- \| --- \| | Cell Signaling Technology | #14472 | 1:1000 (WB) |
| N-cadherin | Cell Signaling Technology | #13116 | 1:1000 (WB) |
| N-catenin | Cell Signaling Technology | #2163 | 1:1000 (WB) |
| Cleaved caspase-9 | Cell Signaling Technology | #7237 | 1:1000 (WB) |
| Cleaved caspase-7 | Cell Signaling Technology | #9491 | 1:1000 (WB) |
| Cleaved caspase-3 | Cell Signaling Technology | #9661 | 1:1000 (WB) |
| Cleaved PARP | Cell Signaling Technology | #5625 | 1:1000 (WB) |
| Caspase-9 | Cell Signaling Technology | #9508 | 1:1000 (WB) |
| Caspase-7 | Cell Signaling Technology | #9492 | 1:1000 (WB) |
| Caspase-3 | Cell Signaling Technology | #9662 | 1:1000 (WB) |
| PARP | Cell Signaling Technology | #9532 | 1:1000 (WB) |
| phospho-Akt | Cell Signaling Technology | #4060S | 1:1000 (WB) |
| phospho-GSK-3β | Cell Signaling Technology | #9322 | 1:1000 (WB) |
| phospho-MTOR | Cell Signaling Technology | #5536 | 1:1000 (WB) |
| Akt | Cell Signaling Technology | #9272 | 1:1000 (WB) |
| GSK-3β | Cell Signaling Technology | #9315 | 1:1000 (WB) |
| MTOR | Cell Signaling Technology | #2972 | 1:1000 (WB) |
| GAPDH | Cell Signaling Technology | #2118 | 1:1000 (WB) |
| LaminA/C | Cell Signaling Technology | #4777 | 1:1000 (WB) |
| Ki67 | Cell Signaling Technology | #9449 | 1:1000 (WB) |
