# Supplementary material for: TMEM65 promotes gastric tumorigenesis by targeting YWHAZ to activate PI3K-Akt-mTOR pathway and is a therapeutic target
Source: Oncogene. 2024 Feb 10;43(13):931–43. doi: 10.1038/s41388-024-02959-9 (PMC10959749; doi:10.1038/s41388-024-02959-9)
Supplement: Supplementary file 3 — Supplementary method [file 41388_2024_2959_MOESM3_ESM.docx]

**Protein extraction and Western blotting**

Total Protein was extracted from cells using RIPA lysis buffer. Proteins were separated on SDS-polyacrylamide gel electrophoresis and transferred onto PVDF membrane. After BSA blocking, the protein-loading membrane was incubated with the primary antibody and secondary antibody. The antibodies used in this study were listed in Supplementary **Table S4**.

**Cell proliferation, colony formation assays**

Cells were plated in 96-well plates at 1,000 cells per well in complete DMEM and used 3-(4,5-dimethylthiazol-2-yl)-2,5-diphenyltetrazolium bromide (MTT) assay (Sigma-Aldrich). After culturing for four hours, use a microplate reader to measure the wavelength of 570 nm. For colony formation assay, cells were plated in 6-well plates at 1,000 cells per well in complete DMEM. Medium was changed every 2 to 3 days. At the endpoint, cells were stained with 0.1% Crystal violet and the number of colonies consisting of >50 cells were counted.

**Wound-healing assay**

Confluent cultures in 6-well plates were scratched with sterile P-200 pipette tips, washed, and cultured in DMEM with free FBS. Cells were photographed after 0, 24, and 48 hours, respectively. The cells migrated across the gap wound were observed and documented using an inverted microscope. Distance of the gap was quantified using Image J.

**Matrigel invasion assays**

For the “Transwell” invasion assays, 3×10^4^ cells with applied genetic modification in 100 μL serum-free medium were seeded onto the upper chamber of Transwell filter (Corning, 354480, Shanghai, China). In the lower chamber, 600 μL complete medium containing 20% FBS was added. After incubation, cells in the lower surface were fixed by methanol, stained with 1% crystal violet, and visualized under a microscope. Three random views were included to calculate the average number of invaded cells.

**Cell cycle and apoptosis analyses**

AGS cells that were stably transfected with TMEM65 or empty vector were plated in a 6-well plate, while MKN74 and MKN7 cells were transfected with siTMEM65 or siNC. After 24h of transfection, the cells were collected and fixed in ice-cold 70% ethanol for 24h before staining with 50μg/ml propidium iodide (BD Biosciences, Franklin Lakes, NJ). The cells were sorted by BD AccuriTM C6 (BD Biosciences), and cell cycle distributions were analyzed using the ModFitLT 5.0 software (Verity Software House, Topsham, ME). All experiments were conducted three times in triplicates. Cells were plated in 6-well plates and serum-starved overnight. Annexin V Apoptosis Detection Kit APC (Invitrogen, Thermo Fisher) was used to determine cell apoptosis. The experiments were conducted three times in triplicates.

**Immunohistochemistry staining**

Immunohistochemistry for TMEM65 was performed on paraffin sections using anti-TMEM65 antibody (ab236861, Abcam) (1:200). The extent of TMEM65 staining was assessed by the areas of focal positivity of nuclei staining. Immunohistochemistry for TMEM65, Ki-67 was also performed on paraffin slides of mouse xenograft tumors and gastric cancer tumor tissues using anti-TMEM65, anti-Ki-67 (**Table S4**).

**Dual luciferase reporter assay**

Briefly, cells were seeded into a 24-well plate and co-transfected with FOXO reporter and Renilla (internal control) reporter. The Firefly and Renilla luminescence were measured by the dual-luciferase reporter assay system (E1910, Promega). Reporter activity was determined as the ratio of Firefly to Renilla luciferase activity.
